# Antillatoxin is a sodium channel activator that displays unique efficacy in heterologously expressed rNa_v_1.2, rNa_v_1.4 and rNa_v_1.5 alpha subunits

**DOI:** 10.1186/1471-2202-11-154

**Published:** 2010-12-14

**Authors:** Zhengyu Cao, William H Gerwick, Thomas F Murray

**Affiliations:** 1 Creighton University, School of Medicine, Department of Pharmacology, Omaha, NE, 68178, USA; 2 Center for Marine Biotechnology and Biomedicine, Scripps Institution of Oceanography, University of California at San Diego, La Jolla, California, 92093-0212, USA; 3 Department of Molecular Biosciences, School of Veterinary Medicine, University of California at Davis, Davis, California, 95616, USA

## Abstract

**Background:**

Antillatoxin (ATX) is a structurally unique lipopeptide produced by the marine cyanobacterium *Lyngbya majuscula*. ATX activates voltage-gated sodium channel α-subunits at an undefined recognition site and stimulates sodium influx in neurons. However, the pharmacological properties and selectivity of ATX on the sodium channel α-subunits were not fully characterized.

**Results:**

In this study, we characterized the pharmacological properties and selectivity of ATX in cells heterologously expressing rNa_v_1.2, rNa_v_1.4 or rNa_v_1.5 α-subunits by using the Na^+ ^selective fluorescent dye, sodium-binding benzofuran isophthalate. ATX produced sodium influx in cells expressing each sodium channel α-subunit, whereas two other sodium channel activators, veratridine and brevetoxin-2, were without effect. The ATX potency at rNa_v_1.2, rNa_v_1.4 and rNa_v_1.5 did not differ significantly. Similarly, there were no significant differences in the efficacy for ATX-induced sodium influx between rNa_v_1.2, rNa_v_1.4 and rNa_v_1.5 α-subunits. ATX also produced robust Ca^2+ ^influx relative to other sodium channel activators in the calcium-permeable DEAA mutant of rNa_v_1.4 α-subunit. Finally, we demonstrated that the 8-demethyl-8,9-dihydro-antillatoxin analog was less efficacious and less potent in stimulating sodium influx.

**Conclusions:**

ATX displayed a unique efficacy with respect to stimulation of sodium influx in cells expressing rNa_v_1.2, rNa_v_1.4 and rNa_v_1.5 α-subunits. The efficacy of ATX was distinctive inasmuch as it was not shared by activators of neurotoxin sites 2 and 5 on VGSC α-subunits. Given the unique pharmacological properties of ATX interaction with sodium channel α-subunits, decoding the molecular determinants and mechanism of action of antillatoxin may provide further insight into sodium channel gating mechanisms.

## Background

**Figure 1:**
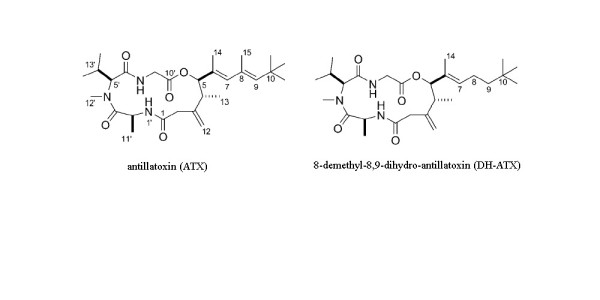
**The chemical structures of antillatoxin (ATX) and 8-demethyl-8,9-dihydro-antillatoxin (DH-ATX)**.

Marine cyanobacteria represent a particularly rich source of structurally novel and biologically active natural products [1]. The range in biological activity is vast and includes compounds that disrupt cell division [2], inhibit microtubule assembly [3], inhibit angiogenesis and promote actin polymerization [4], and block [5] or activate [6] sodium channels. Marine cyanobacteria produce an array of bioactive secondary metabolites that include peptide, polyketide, terpenoid and alkaloid structures. Antillatoxin (ATX, Figure 1) is a structurally unique lipopeptide purified from the pantropical marine cyanobacterium *Lyngbya majuscula *[7]. Blooms of *L. majuscula *have been associated with adverse effects on human health. These adverse effects include respiratory irritation, eye inflammation and sever contact dermatitis in exposed fishermen and swimmers [8].

ATX has been demonstrated to be among the most ichthyotoxic metabolites isolated to date from a marine microalga and is exceeded in potency only by the brevetoxin-1 [7]. ATX has been shown to be neurotoxic in primary cultures of rat cerebellar granule cells [9]. This neurotoxic effect was antagonized by both the sodium channel antagonist tetrodotoxin (TTX) and the N-methyl-D-aspartic acid (NMDA) receptor antagonists, MK-801 and dextrorphan [9]. This profile for ATX toxicity in the rat cerebellar granule cells is therefore similar to that of other voltage-gated sodium channel (VGSC) activators such as brevetoxins [10] suggesting that VGSCs may serve as a molecular target for ATX. Direct evidence for ATX interaction with VGSC was derived from the demonstration of stimulation of [^3^H]batrachotoxin binding and sodium influx by ATX in cultured neurons [11,12]. The precise recognition site for ATX on the VGSC, however, remains to be delineated.

Characterization of the four ATX stereoisomers (all possible C-4 and C-5 diastereomers) has revealed that the preferred stereochemistry for the neuropharmacologic actions of ATX is the (4R, 5R)-isomer [13]. In addition to its stereochemistry, the twisted side chain of ATX has also been demonstrated to be important for its toxicity in neuro-2a mouse neuroblastoma cells. Two ATX analogs, 8-demethyl-antillatoxin and 8-demethyl-8,9-dihydro-antillatoxin (DH-ATX, Figure 1) were shown to be respectively 244- and 27-fold less potent than ATX in producing toxicity in neuro-2a mouse neuroblastoma cells [14].

VGSCs are responsible for the rapid influx of sodium that underlies the rising phase of the action potential in electrically excitable cells including neurons. Sodium channels are composed of voltage-sensing and pore-forming elements in a single protein complex of one principal α-subunit of 220-260 kDa and one or two auxiliary β-subunits which alter the channel physiological properties and subcellular localization [15]. Presently, nine functional VGSC α-subunit isoforms have been described, giving rise to nine sodium channel subtypes termed Na_v_1.1-Na_v_1.9 [16]. VGSC subtypes can be discriminated pharmacologically based on their sensitivity to TTX. Na_v_1.1, 1.2, 1.3, 1.4, 1.6 and 1.7 are sensitive to low nanomolar concentrations of TTX whereas Na_v_1.5 and 1.9 are relatively insensitive to TTX with IC_50 _values in the low micromolar range. Na_v_1.8 is resistant to a concentration of 10 μM TTX. The expression of sodium channel α-subunits is tissue-dependent. Na_v_1.2, 1.4 and 1.5 α-subunits are primarily expressed in the brain, skeletal muscle and cardiac muscle, respectively [16]. VGSCs represent the molecular target for a broad range of potent neurotoxins that bind to at least six distinct receptor sites on the sodium channel α-subunit and affect the two major properties of sodium channels, namely ion permeation and gating [15]. Due to their high affinity and specificity, these neurotoxins serve as important tools to explore the structure and function of VGSCs.

In this study, we characterized the action of ATX on heterologously expressed rNa_v_1.2, rNa_v_1.4 and rNa_v_1.5 α-subunits by measuring sodium influx using a sodium-sensitive fluorescent dye, sodium-binding benzofuran isophthalate (SBFI). We found that ATX displayed unique efficacy as compared to other reference VGSC activators, veratridine and brevetoxin-2 (PbTx-2), in heterologously expressed rNa_v_1.2, rNa_v_1.4 and rNa_v_1.5 α-subunits. These data demonstrate that ATX displays unique pharmacological properties in cells heterologously expressing VGSC α-subunits.

## Results

### ATX produces sodium influx in heterologously expressed rNa_v_1.2, rNa_v_1.4 and rNa_v_1.5 α-subunits

**Figure 2:**
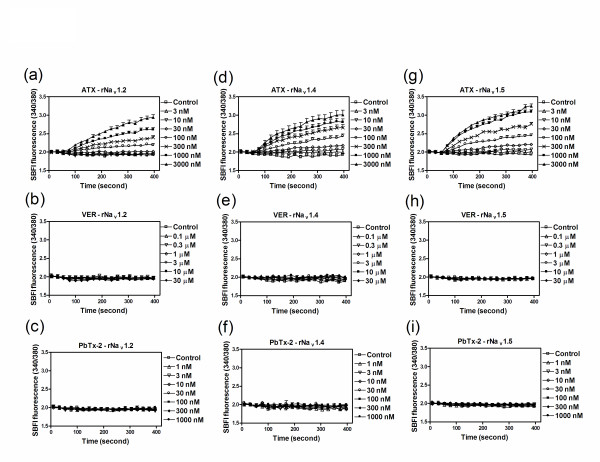
**Time-response relationships for ATX, veratridine and PbTx-2 stimulation of sodium influx in cells heterologously expressing rNa**_**v**_**1.2 (a-c), rNa**_**v**_**1.4 (d-f) and rNa**_**v**_**1.5 (g-i) α-subunits**. Each data point represents the mean ± SEM from at least two different cultures performed in triplicate.

**Figure 3:**
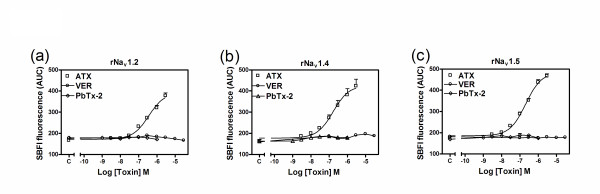
**Concentration-response relationships for ATX, veratridine and PbTx-2 stimulation of sodium influx in heterologously expressed rNa**_**v**_**1.2 (a), rNa**_**v**_**1.4 (b) and rNa**_**v**_**1.5 (c) α-subunits**. Each data point represents mean ± SEM from at least two different cultures performed in triplicate.

We have previously demonstrated that ATX is a sodium channel activator with an undefined recognition site on the VGSC [11]. Recently, ATX has also been demonstrated to stimulate sodium influx in neocortical neurons with a high efficacy comparable to that of the neurotoxin site 2 agonist, batrachotoxin and the neurotoxin site 5 agonist, PbTx-2 [12]. To further characterize this structurally novel toxin, we examined the influence of ATX on sodium influx in heterologously expressed rNa_v_1.2, rNa_v_1.4 and rNa_v_1.5 α-subunits. In preliminary experiments we established that ATX was not toxic to these cell lines heterologously expressing sodium channel α-subunits (Additional file 1). As depicted in Figure 2, ATX produced a rapid and concentration-dependent sodium influx in rNa_v_1.2-CHL 1610, rNa_v_1.4-HEK 293 and rNa_v_1.5-CHL 1610 cells. In contrast neither veratridine, a sodium channel neurotoxin 2 activator nor PbTx-2, a neurotoxin site 5 agonist, were capable of stimulating sodium influx in cells heterologously expressing these sodium channel α-subunits (Figure 2). The concentration-response relationships for ATX-induced sodium influx in rNa_v_1.2-CHL 1610, rNa_v_1.4-HEK 293 and rNa_v_1.5-CHL 1610 cells depicted in Figure 3 indicate that the potencies for ATX did not differ significantly. The EC_50 _values for ATX in rNa_v_1.2, rNa_v_1.4 and rNa_v_1.5 expressing cells are listed in Table 1. The maximal response for ATX-induced sodium influx in rNa_v_1.5-CHL 1610 cells was somewhat greater than that in rNa_v_1.2-CHL 1610 and rNa_v_1.4-HEK 293 cells (Figure 3). The rank order of maximal response for ATX-stimulated sodium influx was rNa_v_1.5-CHL 1610 > rNa_v_1.4-HEK 293 > rNa_v_1.2-CHL 1610.

**Table 1:** Potency and relative efficacy for ATX, DH-ATX, veratridine and PbTx-2-induced sodium influx in rNav1.2, rNav1.4 and rNav1.5 α-subunits

	**rNa**_**v**_**1.2**		**rNa**_**v**_**1.4**		**rNa**_**v**_**1.5**	
	**EC**_**50 **_**(nM) (95% CI)**	Efficacy	**EC**_**50 **_**(nM) (95% CI)**	Efficacy	**EC**_**50 **_**(nM) (95% CI)**	Efficacy
ATX	376.5 (258.5-548.3)	1	164.6 (87.4-309.9)	1	205.1 (155.8-270.1)	1
DH-ATX	1,129 (384.3-3,314)	0.32	644.1 (293.7-1,412)	0.33	595.1 (173.6-2,040)	0.25
Veratridine	N/A	0	N/A	0	N/A	0
PbTx-2	N/A	0	N/A	0	N/A	0

### ATX produces minimal sodium influx in wild type HEK 293 and CHL 1610 Cells

**Figure 4:**
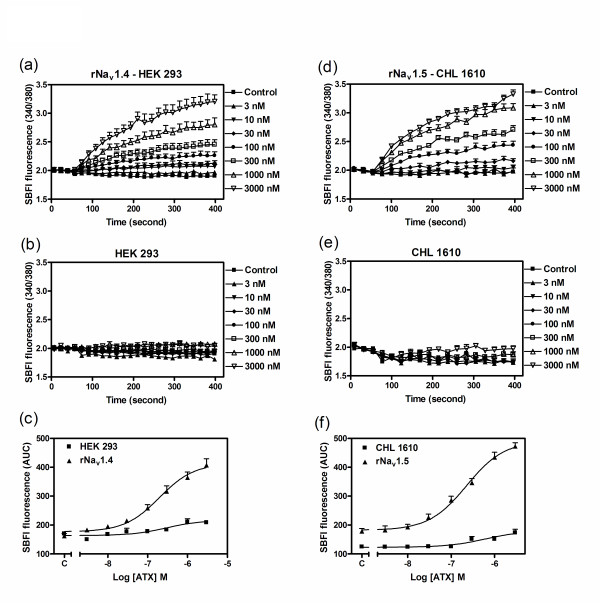
**Influence of ATX on sodium influx in HEK 293 and rNa**_**v**_**1.4-HEK 293, or CHL 1610 and rNa**_**v**_**1.5-CHL 1610 cells**. Time-response relationships for ATX induced sodium influx in rNa_v_1.4-HEK 293 (a), HEK 293 (b) rNa_v_1.5-CHL1610 (d) and CHL1610 cells (e); Concentration-response relationships for ATX-induced sodium influx in rNa_v_1.4-HEK 293 and HEK 293 (c), and CHL 1610 and rNa_v_1.5-CHL 1610 cells (f). These data were obtained from at least two different cultures performed in triplicate.

To demonstrate that the sodium influx observed in response to ATX exposure was a consequence of activation of rNa_v_1.2, rNa_v_1.4 and rNa_v_1.5 α-subunits, we examined the influence of ATX in the HEK 293 and CHL 1610 parent cell lines. ATX produced minimal sodium influx in both HEK 293 and CHL 1610 cells (Figure 4). These data therefore indicate that the ATX-induced sodium influx observed in cells stably expressing VGSC α-subunits was primarily a consequence of activation of rNa_v_1.2, rNa_v_1.4 and rNa_v_1.5. The marginal sodium influx induced by ATX in wild type HEK 293 likely resulted from activation of endogenously expressed sodium channels [16,17]. Although there are no reports of the expression of sodium channel α-subunits in CHL 1610 cells, our data suggest that these cells may also express low levels of endogenous sodium channel α-subunits.

### TTX antagonism of ATX-induced sodium influx in rNa_v_1.2-CHL 1610, rNa_v_1.4-HEK 293 and rNa_v_1.5-CHL 1610 cells

**Figure 5:**
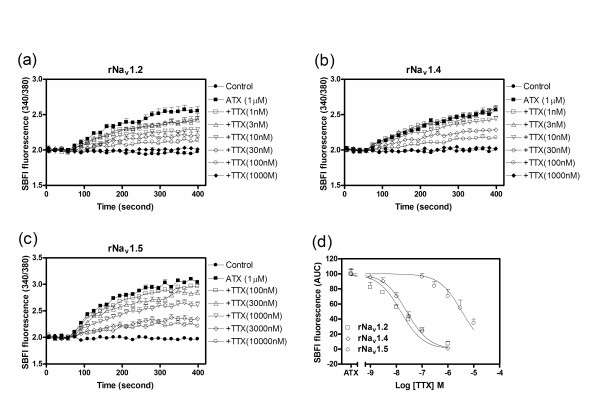
**TTX antagonizes ATX-induced sodium influx in cells expressing rNa**_**v**_**1.2, rNa**_**v**_**1.4 and rNa**_**v**_**1.5 α-subunits**. Time-response for TTX antagonism of ATX-induced sodium influx in cells expressing rNa_v_1.2 (a), rNa_v_1.4 (b) and rNa_v_1.5 (c) α-subunits; (d) Concentration-response for TTX antagonism of ATX-induced sodium influx in cells expressing rNa_v_1.2, rNa_v_1.4 and rNa_v_1.5 α-subunits. Each data point represents the mean ± SEM from two different cultures performed in triplicate.

To further confirm the role of rNa_v_1.2, rNa_v_1.4 and rNa_v_1.5 α-subunits in ATX-induced sodium influx, we examined the influence of TTX on the response to ATX. TTX pretreatment produced a concentration-dependent inhibition of ATX-induced sodium influx in rNa_v_1.2-CHL 1610, rNa_v_1.4-HEK 293 and rNa_v_1.5-CHL 1610 cells with IC_50 _values of 14.8 nM (11.2-19.8, 95% CI), 25.2 nM (20.1-31.7 nM, 95% CI) and 3.47 μM (2.33-5.15, 95% CI), respectively (Figure 5). These inhibitory potencies of TTX are consistent with the expected sodium channel pharmacology in that rNa_v_1.2 and rNa_v_1.4 are TTX sensitive with IC_50 _values in the nanomolar range whereas rNa_v_1.5 is relatively insensitive with an IC_50 _value in the low micromolar range [18].

### Relative densities of rNa_v_1.5, rNa_v_1.2 and rNa_v_1.4 α-subunits expressing cells

**Figure 6:**
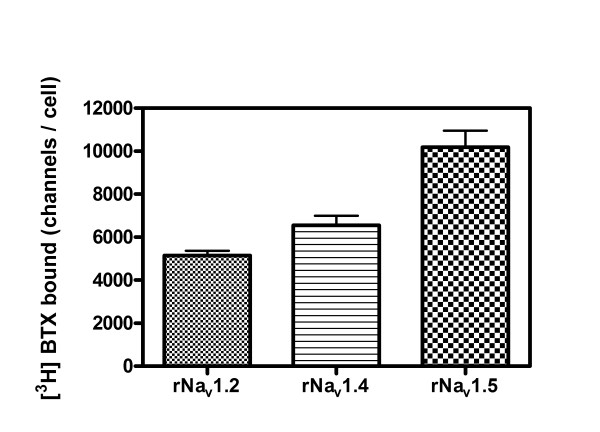
**The relative expression levels of rNa**_**v**_**1.2, rNa**_**v**_**1.4 and rNa**_**v**_**1.5 α-subunits determined by [**^**3**^**H]BTX binding to intact cells**. The expression levels of sodium channels are given in units of channels/cell. Each data point represents mean ± SEM for five determinations.

Given the modest differences in the maximal responses for ATX in rNa_v_1.2-CHL 1610, rNa_v_1.4-HEK 293 and rNa_v_1.5-CHL 1610 cells, we performed a whole-cell [^3^H]BTX binding assay to determine the relative density of these sodium channel α-subunits, which may contribute to the distinct maximal response for ATX action. Assuming equivalent [^3^H]BTX affinities for rNa_v _1.2, 1.4 and 1.5, a single radioligand concentration may be used to estimate the relative densities of binding sites. [^3^H]BTX is a selective probe for neurotoxin site 2 on the VGSC α-subunits [19]. This ligand interacts preferentially to the active or open conformation of the channel, and the specific binding is sensitive to conformational changes induced by the binding of neurotoxins at other sites on the sodium channel α-subunit. Inasmuch as the specific binding of [^3^H]BTX is dramatically increased by the allosteric effects of deltamethrin and brevetoxin, we performed the [^3^H]BTX binding assay in the presence of these positive allosteric modulators. The specific binding of [^3^H]BTX (1.8 nM) to cells expressing either rNa_v_1.2, rNa_v_1.4 or rNa_v_1.5 α-subunits indicated that rNa_v_1.5 had the highest expression level (10,187 ± 763 channels/cell, mean ± SEM) followed by rNa_v_1.4 (6,560 ± 437 channels/cell) and rNa_v_1.2 (5146 ± 223 channels/cell) (Figure 6). The maximal sodium influx evoked by ATX paralleled these relative densities of VGSC α-subunits (Additional file 2). These data indicate that the modest difference in maximum responses to ATX in the three cell lines is primarily a function of the relative densities of α-subunits.

### Positive allosteric interaction between ATX and PbTx-2 binding sites on the rNa_v_1.4 α-subunit

**Figure 7:**
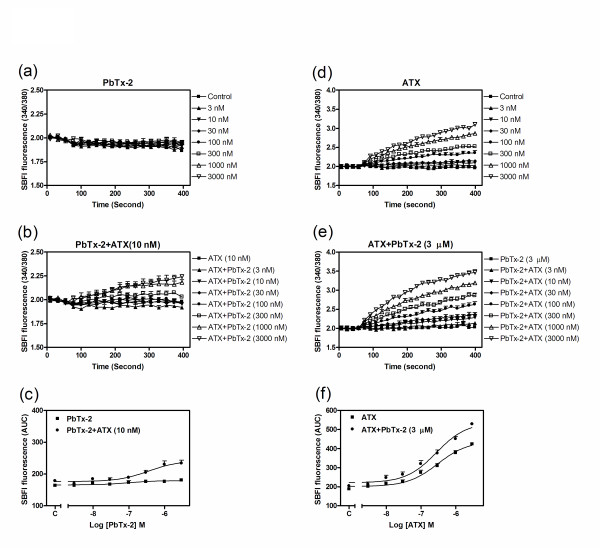
**Reciprocal allosteric interaction between ATX and neurotoxin site 5 in cells expressing rNa**_**v**_**1.4 α-subunits**. (a), (b), Time-response relationships for PbTx-2 induced sodium influx in the absence and presence of 10 nM of ATX in cells expressing rNa_v_1.4 α-subunits; (c), Concentration-response relationships for PbTx-2-induced sodium influx in the absence and presence of 10 nM of ATX; (d), (e), Time-response relationships for ATX-induced sodium influx in the absence and presence of 3 μM of PbTx-2 in cells expressing rNa_v_1.4 α-subunits; (f), Concentration-response relationships for ATX-induced sodium influx in the absence and presence of 3 μM of PbTx-2. These data were repeated twice in triplicate with similar results.

ATX binds to an undefined site on the sodium channel α-subunit and this site is allosterically coupled to neurotoxin sites 2 and 5 [11]. To examine the allosteric interaction between the ATX binding site and neurotoxin site 5 in rNa_v_1.4-HEK 293 cells, we evaluated the concentration-response relationships for PbTx-2 to stimulate sodium influx in the absence or presence of 10 nM ATX. While PbTx-2 alone had no effect on sodium influx, in the presence of a subthreshold concentration of ATX (10 nM) PbTx-2 concentration-dependently increased sodium influx in rNa_v_1.4-HEK 293 cells (Figure 7a, b &7c). To ascertain the reciprocal nature of this positive allosteric interaction, we also examined the influence of a fixed concentration of PbTx-2 (3 μM) on ATX-induced sodium influx in rNa_v_1.4-HEK 293 cells. PbTx-2 (3 μM), which alone had no effect, significantly increased the maximal response to ATX-induced sodium influx from an area under the curve (AUC, fluorescence × time) value of 439.4 (413.4-465.4, 95%CI) to 537.9 (501.4-574.4, 95%CI) (Figure 7d, e, f). These data demonstrate a balanced positive allosteric interaction between the ATX binding site and neurotoxin site 5.

### DH-ATX is a partial agonist in cells expressing rNa_v_1.2, rNa_v_1.4 and rNa_v_1.5 α-subunits

**Figure 8:**
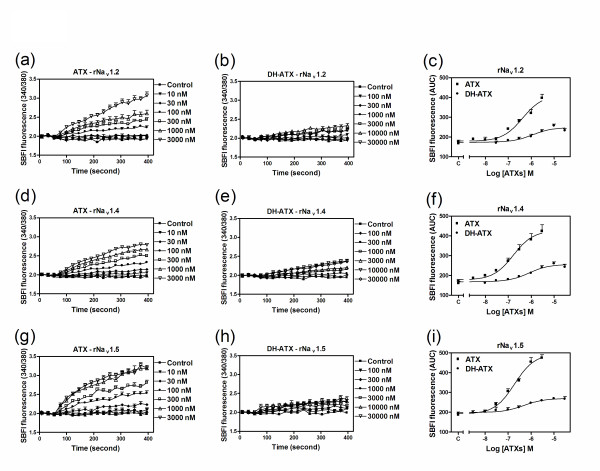
**Influence of ATX and DH-ATX on the stimulation of sodium influx in cells expressing rNa**_**v**_**1.2 (a, b), rNa**_**v**_**1.4 (d, e) and rNa**_**v**_**1.5 (g, h) α-subunits**. Time-response relationships for ATX and DH-ATX-induced sodium influx in cells expressing rNa_v_1.2 (a, b), rNa_v_1.4 (d, e) and rNa_v_1.5 (g, h) α-subunits; Concentration-response relationships for ATX and DH-ATX-induced sodium influx in cells expressing Na_v_1.2 (c), rNa_v_1.4 (f) and rNa_v_1.5 α-subunits (i). Each data point represents mean ± SEM from two different cultures performed in triplicate.

DH-ATX is a recently synthesized ATX analog [14]. DH-ATX produces toxicity in neuro-2a neuroblastoma cells with an EC_50 _of 1.2 μM, which is less potent than ATX in the same system [14]. To expand the structure-activity relationship information for this analog, we examined the effects of DH-ATX on the stimulation of sodium influx in rNa_v_1.2-CHL 1610, rNa_v_1.4-HEK 293 and rNa_v_1.5-CHL 1610 cells. DH-ATX produced concentration-dependent stimulation of sodium influx in all three cell lines, and was 2.5 - 4-fold less potent than ATX (Figure 8). Additionally, the maximal response of DH-ATX on sodium influx was lower than that of ATX in cells expressing rNa_v_1.2, rNa_v_1.4 and rNa_v_1.5 α-subunits (Figure 8). These data demonstrate that DH-ATX acts as a partial agonist in cells heterologously expressing sodium channel α-subunits. The EC_50 _values and relative efficacy for DH-ATX stimulation of sodium influx in cells expressing rNa_v_1.2, rNa_v_1.4 and rNa_v_1.5 α-subunits are listed in Table 1.

### ATX and DH-ATX produce Ca^2+ ^influx in DEAA-HEK 293 cells

**Figure 9:**
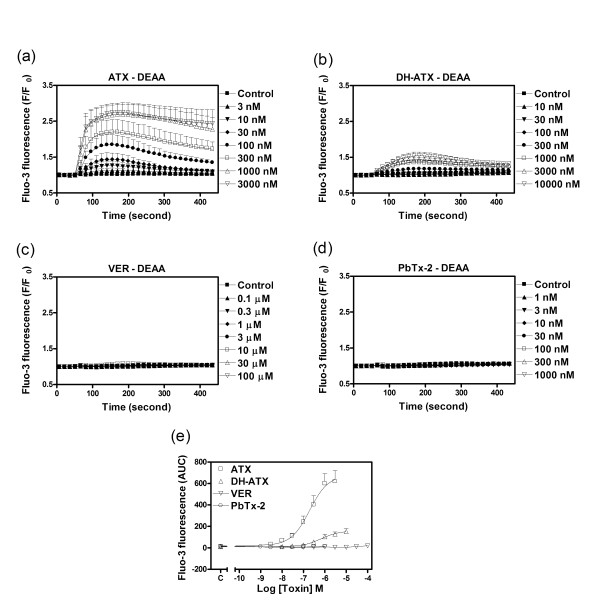
**Influence of ATX, DH-ATX, veratridine and PbTx-2 on the stimulation of Ca**^**2+ **^**influx in DEAA-HEK 293 cells**. Time-response relationships for ATX (a), DH-ATX (b), veratridine (c) and PbTx-2 (d) stimulation of Ca^2+ ^influx in DEAA-HEK 293 cells; (e), Concentration-response relationships for ATX, DH-ATX, veratridine and PbTx-2 stimulation of Ca^2+ ^influx in DEAA-HEK 293 cells. These data were repeated four times in duplicate with similar results.

Sodium channel cation permeability is intimately related to the conserved motif of four amino acid residues located near the outer vestibule of the pore. These residues form the DEKA locus in sodium channel α-subunits. Native Na^+ ^channels are virtually impermeable to Ca^2+^. Mutation of the conserved Lys (K) residue in domain III to Ala (A), (DEAA) produces a channel that is permeable to Ca^2+ ^[20]. We used cells expressing the rNa_v_1.4 DEAA mutation to explore the influence of sodium channel activators on Ca^2+ ^influx. ATX produced a robust and concentration-dependent Ca^2+ ^influx with an EC_50 _value of 165.4 nM (122.9-222.5, 95% CI) that was similar to its potency on sodium influx in rNa_v_1.4-HEK 293 cells. The ATX analog, DH-ATX stimulated Ca^2+ ^influx with an EC_50 _value of 679.6 nM (279.8-1,651, 95%CI). The relative efficacy for DH-ATX-induced Ca^2+ ^influx was 0.24 which was similar to its relative efficacy in stimulating sodium influx in native rNa_v_1.4 cells (0.33, Table 1). Again, these data demonstrate that DH-ATX is a partial agonist in cells expressing VGSC α-subunits. Similar to the lack of response on sodium influx in rNa_v_1.4-HEK 293 cells, veratridine and PbTx-2 were all without effect on the stimulation of Ca^2+ ^influx in DEAA-HEK 293 cells (Figure 9).

## Discussion

Antillatoxin is a structurally unique lipopeptide characterized by a large number of methyl groups, including a rare *tert*-butyl group [7]. ATX binds to a site on the sodium channel α-subunit that remains to be defined and stimulates sodium influx in cerebellar granule and neocortical neurons [11,12]. Veratridine is a steroid-derived alkaloid purified from the plant *Liliaceae *family. Veratridine specifically interacts with neurotoxin site 2 and preferentially binds to the activated state of sodium channels. Veratridine produces persistent channel activation by inhibiting sodium channel inactivation and shifting the voltage dependence of activation to more negative potentials [15,21]. PbTx-2, a lipid soluble polyether neurotoxin, is produced by the marine dinoflagellate *Karenia brevis*. PbTx-2 specifically binds to neurotoxin site 5 of sodium channel α-subunits and shifts the activation potential to more negative values and inhibits channel inactivation [15].

In the present experiments, we demonstrated that ATX produced an efficacious stimulation of sodium influx in heterologously expressed rNa_v_1.2, rNa_v_1.4 and rNa_v_1.5 α-subunits. Although whole-cell patch-clamp is an elegant and useful method to study ion channel function with temporal resolution capable of tracking the millisecond kinetics of activation and inactivation, the low throughput of such electrophysiological methods precludes comprehensive assessment of concentration-response relationships as reported in the present study. The SBFI fluorescence method used in our study provided a robust and homogeneous cell population measurement. Our results indicate that ATX acts by binding to the α-subunit of neuronal, cardiac and skeletal muscle sodium channels. ATX also produced robust stimulation of Ca^2+ ^influx in the mutant DEAA-rNa_v_1.4 expressing cells. ATX has similarly been shown to be a high efficacy sodium channel activator in neocortical neurons [12]. The resting membrane potential for HEK 293 cells is relatively depolarized (-35 ± 5 mV) [22]. At this depolarized resting membrane potential most rNa_v_1.4 channels will be in the inactivated state. The efficacious response for ATX on sodium influx in rNa_v_1.4-HEK 293 cells suggests that ATX may either alter the voltage dependence of inactivation or augment the rate of recovery from inactivation. It is also possible that ATX may interact preferentially with the inactivated state of VGSC α-subunits. An inactivated state dependency has been demonstrated previously for the ability of β scorpion toxin to modify VGSC function [23]. Depolarizing stimuli have also been shown to enhance the binding of β scorpion toxin to neuronal cells [24]. These earlier observations demonstrate precedence for preferential interaction of neurotoxins with the inactivated state of VGSC α-subunits.

In contrast to the efficacy of ATX on sodium influx in cells expressing rNa_v_1.2, rNa_v_1.4 and rNa_v_1.5 α-subunits, we found that veratridine and PbTx-2 were without significant effect when administered alone. These toxins were also without effect on the stimulation of Ca^2+ ^influx in DEAA-rNa_v_1.4 expressing cells. In neocortical neurons we have previously demonstrated that veratridine and PbTx-2 produce sodium influx with respective efficacies of 0.67 and 0.81 [12]. The higher sodium channel expression levels in neurons may partly account for these differences in the efficacies of veratridine and PbTx-2 [25]. Whereas neurons and HEK cells [26] express sodium channel β-subunits, CHL 1610 cells do not [27]. Sodium channel β-subunits regulate sodium channel α-subunit function at multiple levels including mRNA expression, channel stabilization/trafficking and direct channel modulation [28]. The β1 subunit has been shown to alter toxin sensitivity of VGSC α-subunits reconstituted in phospholipid vesicles [29], but not in CHO-K1 cells [30]. The role of the absence of the VGSC β1 subunit in the lack of effect of veratridine and PbTx-2 on sodium influx in cells expressing rNa_v_1.2 and rNa_v_1.5 α-subunits remains to be determined. The ability of ATX to stimulate sodium influx in these cell lines is, however, clearly not dependent on the expression of the VGSC β1 subunit. In this regard it is noteworthy that modulation of VGSC function by β scorpion toxin does not depend on the presence of the sodium channel β1 subunit [23]).

Among these cell lines expressing α-subunits, we demonstrated that ATX was somewhat more potent (2-fold) at the skeletal muscle (rNa_v_1.4) and cardiac (rNa_v_1.5) than in the neuronal rNa_v_1.2. The maximal response for ATX-stimulated sodium influx in rNa_v_1.5-CHL 1610 cells was modestly higher than in rNa_v_1.2-CHL 1610 or rNa_v_1.4-HEK 293 cells. The whole cell [^3^H]BTX binding assay, however, demonstrated that these distinct maximal responses were related to the relative sodium channel α-subunit expression levels in these cell lines.

DH-ATX is an ATX analog, which is less potent than ATX in producing toxicity in the neuro-2a mouse neuroblastoma cell line [14]. We have demonstrated that the ability of DH-ATX to produce sodium influx was also less potent than ATX in cells expressing rNa_v_1.2, rNa_v_1.4 and rNa_v_1.5 α-subunits. Additionally, we have demonstrated that the efficacy of DH-ATX in the stimulation of sodium influx was lower than that of ATX in these cells. A similar profile of both lower potency and relative efficacy was observed when measuring Ca^2+ ^influx in DEAA-HEK 293 cells. These data demonstrate that the twisted side chain of ATX is important for the interaction with sodium channel α-subunits. Lipid-soluble toxins at neurotoxin sites 2 and 5 have been shown to affect channel gating [31,32]. Catterall has provided evidence that the site 2 toxins batrachotoxin, aconitine and veratridine act as full or partial agonists [31,32]. We have previously demonstrated that a group of site 5 ligands, including both naturally occurring and semi-synthetic brevetoxin analogs, produce distinct maximal responses on sodium influx in neocortical neurons [12]. The lower efficacy of DH-ATX relative to ATX suggests that the partial agonism observed previously for neurotoxin site 2 and 5 ligands generalizes to the ATX site on VGSC α-subunits.

The lipid-soluble toxins acting at neurotoxin receptor sites 2 and 5 have been characterized as allosteric modulators of sodium channel function [15]. These toxins bind at topologically distinct sites that favor the open state of the sodium channel and display complex allosteric interactions. ATX has been demonstrated to allosterically stimulate [^3^H]BTX binding in cerebellar granule cells [11]. This enhancement can be further augmented by PbTx-2 suggesting a positive allosteric interaction between neurotoxin site 5 and the ATX site [11]. In the present study we demonstrated that a subthreshold concentration of ATX significantly potentiated PbTx-2-induced sodium influx in rNa_v_1.4 α-subunit expressing cells. PbTx-2 also significantly augmented ATX-induced sodium influx in these cells. These data therefore directly demonstrate the reciprocal nature of the allosteric interaction between the ATX binding site and neurotoxin site 5. This is consistent with the free energy conservation principle for the simultaneous binding of two allosterically coupled ligands in which the binding of ligand A produces upon the binding of ligand B the same effects that the binding of ligand B produces upon the binding of ligand A [33].

## Conclusions

In this study we demonstrate that ATX displays a unique efficacy with respect to stimulation of sodium influx in cells expressing rNa_v_1.2, rNa_v_1.4 and rNa_v_1.5 α-subunits. The efficacy of ATX was distinctive inasmuch as it was not shared by activators of neurotoxin sites 2 and 5. It is possible that, similar to β scorpion toxin interaction with neurotoxin site 4, ATX may interact preferentially with the inactivated state of VGSC α-subunits. We have also demonstrated that the ATX binding site shares with neurotoxin sites 2 and 5 the phenomenon of partial agonism. Finally, we observed a reciprocal allosteric interaction between neurotoxin site 5 and the ATX binding site. Collectively, these data indicate that ATX is a sodium channel gating modifier with unique efficacy in cells heterologously expressing VGSC α-subunits. Defining the molecular determinants and mechanisms of action of ATX may provide further insight into the gating properties of sodium channels.

## Methods

### Cell culture

CHL 1610 cells were cultured in DMEM/F12 with glutamine and supplemented with 5% FBS, 100 units/ml peniclillin and 0.1 mg/ml streptomycine. rNa_v_1.2-CHL 1610 and rNa_v_1.5-CHL 1610 cells were cultured in DMEM/F12 with glutamine and supplemented with 5% FBS, 100 units/ml peniclillin, 0.1 mg/ml streptomycin and 200 μg/ml Geneticin. HEK 293 cells were cultured in DMEM and supplemented with 10% FBS, 100 units/ml peniclillin and 0.1 mg/ml streptomycine. rNa_v_1.4-HEK 293 and DEAA-HEK 293 cells were cultured in DMEM and supplemented with 10% FBS, 100 units/ml peniclillin, 0.1 mg/ml streptomycine, 700 μg/ml geneticin. All cells were grown routinely as monolayers on poly-D-lysine coated T75 flask in an atmosphere of 5% CO_2 _and 37°C.

### Sodium influx assay

The cells were plated onto poly-D-lysine coated 96-well plates at initial densities of 25,000/well (for CHL 1610, rNa_v_1.2-CHL 1610 and rNa_v_1.5-CHL 1610) or 50,000/well (for HEK 293 and rNa_v_1.4-HEK 293 cells) and cultured overnight. The cells were then washed four times with Locke's buffer (in mM: 8.6 HEPES, 5.6 KCl, 154 NaCl, 5.6 Glucose, 1.0 MgCl_2_, 2.3 CaCl_2_, 0.0001 glycine, pH 7.4) using an automated cell washer (Biotek instrument Inc., VT, USA). The background fluorescence of each well was measured and averaged prior to dye loading. Cells were then incubated for 1 h at 37°C with dye loading buffer (50 μl/well) containing 10 μM SBFI-AM, 0.04% pluronic acid F-127 and 2.5 mM probenecid in Locke's buffer. After 1 h incubation in dye loading buffer, cells were washed twice with Locke's buffer supplemented with 2.5 mM probenecid, leaving a final volume of 150 μl in each well. The plate was then transferred to the chamber of a FLEXstation™ II (Molecular Devices, Sunnyvale, CA, USA). Cells were excited at 340 nm and 380 nm and Na^+^-bound SBFI emission was detected at 505 nm. Fluorescence readings were taken once every 5 s for 60 s to establish the baseline and then 50 μl of neurotoxin containing solution (4x) was added to each well from the compound plate at a rate of 26 μl/s. The fluorescence signals were recorded for an additional 5.5 min after addition of toxins. All the experiments were performed at 37°C.

### Calcium influx assay

The DEAA-HEK cells were planted onto poly-D-lysine coated 96-well plates at an initial density of 50,000/well and cultured overnight. Briefly, the growth medium was removed and replaced with dye loading buffer (100 μl/well) containing 4 μM fluo-3, 0.04% pluronic acid F-127 and 2.5 mM probenecid in Locke's buffer. After 1 h incubation in dye loading buffer, cells were washed four times in fresh Locke's buffer supplemented with 2.5 mM probenecid (200 μl/well) and transferred to the plate chamber of a FLEXstation™ II (Molecular Devices, Sunnyvale, CA, USA). The final volume of Locke's buffer in each well was 150 μl. The cells were excited at 488 nm and Ca^2+ ^bound fluo-3 emission at 535 nm was recorded. Fluorescence readings were taken every 2 seconds for 60 s to establish the baseline. Different concentrations (4x) of toxins were added to the cells from a compound plate in a volume of 50 μl at a rate of 26 μl/s and the fluorescence signals were recorded for an additional 6 min. All the experiments were performed at 37°C.

### Whole cell binding assay

The cells were plated in poly-D-lysine coated 6-well plate at densities of 300,000/well (for rNa_v_1.2-CHL 1610 and rNa_v_1.5-CHL 1610) or 600,000/well (for rNa_v_1.4-HEK 293 cells) and cultured overnight. Cells were first rinsed three times with 1 ml of buffer A containing 140 mM choline chloride, 5 mM KCl, 1.8 mM CaCl_2_, 0.8 mM MgSO_4 _and 10 mM Hepes (pH 7.4 with 1 M Tris). Cells were then incubated with 1 ml of buffer B (buffer A plus 2 mg/ml BSA) containing 1.8 nM [^3^H]BTX, 300 nM PbTx-3 and 10 μM deltamethrin for 3 h. After incubation, cells were rinsed three times with 1 ml of buffer A before lysis with 500 μl of 1% Triton X-100. A 400 μl aliquot of the resulting lysate was collected, and [^3^H]BTX bound was measured by liquid scintillation counter. Nonspecific binding of [^3^H]BTX was defined as that occurring in the presence of 10 μM hoiamide A. Hoiamide A is a recently described sodium channel neurotoxin site 2 ligand. Hoiamide A has been shown to inhibit [^3^H]BTX binding with an IC_50 _value of 92.8 nM which is 360-fold more potent than the prototypic neurotoxin site 2 agonist, veratridine [6]. Our preliminary results indicated that whole cell assays conducted at room temperature (22°C) provided an optimum signal to noise ratio. Therefore all binding experiments were conducted at 22°C.

### Data analysis

The SBFI raw emission data at each excitation wavelength were exported to an Excel work sheet and corrected for background fluorescence. The SBFI fluorescence ratios (340/380) or fluo-3 fluorescence (F/F_0_) versus time were analyzed and concentration-response graphs were generated using Graphpad Prism software (Version 4.0, La Jolla, CA, USA). The EC_50 _and maximum responses for ATX and DH-ATX stimulation of sodium or calcium influx were determined by non-linear regression analysis using a three-parameter logistic equation.

### Materials

ATX and DH-ATX were synthesized as described by Okura et al. (2010). Penicillin, streptomycin and heat-inactivated fetal bovine serum (FBS) were obtained from Atlanta Biologicals (Norcross, GA, USA). Poly-D-lysine (molecular weight >300,000), Geneticin, probenecid, Dulbecco's Modified Eagle Medium (DMEM), Dulbecco's Modified Eagle Medium/Nutrient Mixture F-12 (DMEM/F12) and veratridine were from Sigma-Aldrich (St. louis, MO, USA). The fluorescent dye SBFI-AM, fluo-3-AM and pluronic acid F-127 were obtained from Invitrogen Corporation (Carlsbad, CA, USA). Tetrodotoxin (TTX) was purchased from Tocris Cookson, Inc. (Ellisville, MO, USA). [^3^H]batrachotoxin A 20-α-Benzoate ([^3^H]-BTX) was from PerkinElmer (Waltham, MA, USA). Brevetoxin-2 (PbTx-2) was isolated and purified from *K. breves *cultures at the Center for Marine Science at the University of North Carolina (Wilmington, NC). The human embryonic kidney 293 (HEK 293) and Chinese hamster lung (CHL) 1610 cells were from American Type Culture Collection (Manassas, VA, USA). The rNa_v_1.4-HEK 293 and the DEKA locus mutant (DEAA-HEK 293) cell lines were obtained under the license from Dr. E. Moczydlowski (Clarkson University, Potsdam, NY, USA). rNa_v_1.2-CHL 1610 and rNa_v_1.5-CHL 1610 were obtained under the license from Dr. W. Catterall (University of Washington, Seattle, WA).

## Authors' contributions

ZC designed and carried out the experiments, participated in the data analysis and drafted the manuscript. WHG provided the compound. TFM participated in the design the experiments, performed the analysis and drafted the manuscript. All authors read and approved the final manuscript.

## Supplementary Material

Additional file 1 **Lack of toxic effect of ATX in rNa**_**v**_**1.2-CHL 1610, rNa**_**v**_**1.4-HEK 293 and rNa**_**v**_**1.5-CHL 160 cells**. The cells were exposed to 1 μM ATX for 24 h and the medium was collected and subjected to LDH activity measurement as described by Koh and Choi (1987; Koh, JY, and Choi, DW. Quantitative determination of glutamate mediated cortical neuronal injury in cell culture by lactate dehydrogenase efflux assay. *J Neurosci Methods *20:83-90). These data are normalized to percentage of maximal LDH activity resulted from H_2_O cell lysate. Click here for file

Additional file 2 **Correlation between ATX-induced maximal response on sodium influx and the relative channel densities of heterologously expressed rNa**_**v**_**1.2, rNa**_**v**_**1.4 and rNa**_**v**_**1.5 α-subunits**. The displayed regression line was derived from linear regression (r^2 ^= 0.9928). Click here for file
